# GHM-DEIM: An Improved DEIM-Based Framework for Subtle and Scale-Variant Thermal Anomaly Detection in Photovoltaic UAV Infrared Imagery

**DOI:** 10.3390/s26123796

**Published:** 2026-06-14

**Authors:** Jianxiang Li, Lang Yang, Wei Huang, Feng Ren, Jing Hu

**Affiliations:** School of Mathematics and Computer Science, Wuhan Polytechnic University, Wuhan 430040, China; lijianxiang@whpu.edu.cn (J.L.); yanglang425@163.com (L.Y.); huangwei@whpu.edu.cn (W.H.); renfeng2026@163.com (F.R.)

**Keywords:** feature extraction, hypergraph, modulation fusion module, thermal anomaly detection, thermal images, infrared images, photovoltaic systems, solar power

## Abstract

With the increasing demand for low-carbon energy, automated defect detection using unmanned aerial vehicle (UAV)-based thermal inspection has become essential for maintaining the reliability of photovoltaic systems. However, existing methods still suffer from low-contrast thermal imagery, large-scale variations of defects, and subtle thermal anomalies. To address these challenges, this study proposes Grouped-Hypergraph-Modulation DEIM (GHM-DEIM), a robust end-to-end detection framework based on an improved DEIM architecture. Specifically, a grouped multi-scale aggregation attention network is introduced to enhance global thermal perception and recover discriminative features from blurred backgrounds. In addition, an enhanced encoder incorporating a hypergraph-based context encoding mechanism is designed to model high-order non-local relationships and improve feature representation across different defect scales. Furthermore, a modulation fusion module is employed to adaptively refine multi-scale feature responses and suppress environmental noise interference. Extensive experiments conducted on the ThermoSolar-PV and PV-HSD-2025 datasets demonstrate that the proposed method consistently outperforms state-of-the-art detectors, achieving mAP@50 values of 88.6% and 74.2%, respectively, with improvements of 4.7% and 2.9% over the baseline. These results demonstrate the effectiveness and robustness of GHM-DEIM for UAV-based PV thermal defect inspection.

## 1. Introduction

As the demand for low-carbon energy continues to rise, solar power facilities are being deployed at an accelerating pace [1,2]. Among various renewable sources, solar PV systems have witnessed explosive growth in installed capacity over the past decade, owing to their clean nature and ease of deployment [3]. However, as photovoltaic (PV) modules are typically deployed in harsh outdoor environments, they are inevitably susceptible to various defects, such as hot spots, cracks, potential induced degradation, and diode failures [4,5]. Studies indicate that these anomalies not only significantly reduce the power generation efficiency of PV plants, leading to irreversible power loss, but can also induce fire hazards in severe cases, resulting in substantial economic losses and safety risks [6,7]. Therefore, developing efficient and accurate anomaly detection technologies has become an urgent demand in both industry and academia to ensure the long-term stable operation of PV systems and reduce operation and maintenance costs [8].

Traditional inspection of solar PV plants relies primarily on manual on-site patrolling using handheld devices [9]. This approach is not only time-consuming and inefficient but also poses safety risks, particularly for large-scale PV facilities installed on rooftops or in remote areas [10]. With the rapid development of Unmanned Aerial Vehicle (UAV) technology, UAV-based aerial inspection has emerged as a promising alternative [11]. Among various sensor modalities, Infrared Thermography is recognized as one of the most effective Non-Destructive Testing (NDT) techniques [12]. Unlike visible-light cameras, which can only capture superficial physical damages, thermal imaging sensors are capable of perceiving the temperature distribution across the PV module surface. Since internal circuit failures—such as cell cracks or poor interconnect soldering—typically result in localized overheating, these defects manifest as distinct “hot spots” in infrared images [13]. Consequently, these aerial platforms equipped with thermal infrared cameras enable the rapid and large-scale identification of potential anomalies that remain invisible to the eye.

In recent years, automated detection algorithms based on computer vision have gradually replaced traditional methods relying on thresholding and edge detection [14,15]. CNN, represented by the YOLO series [16] and Faster R-CNN [17], have achieved significant success in PV defect detection. However, the convolution operations in CNN are limited by their local receptive fields, often struggling to capture global context information within large-scale PV arrays. Furthermore, YOLO models typically rely on Non-Maximum Suppression (NMS) for post-processing, which can lead to missed detections in scenarios with densely arranged PV modules. To address this, Transformer-based detectors such as DETR [18] and RT-DETR [19] redefine object detection as a set-based prediction problem, thus removing the need for NMS. They apply global self-attention to capture long-range dependencies, enhancing detection stability under dense and complex conditions.

Despite these advancements, applying these models directly to PV thermal imagery remains challenging. Firstly, unlike visible-light images, thermal infrared images typically exhibit low contrast and blurred texture details. Typical backbone networks often fail to extract discriminative thermal features from such data [20]. Secondly, PV anomalies vary drastically in size—ranging from tiny cell-level hot spots to large-area diode failures. Existing feature pyramids often struggle to balance detection performance across these scale variations [21]. Finally, Standard feature fusion mechanisms usually rely on simple direct summation or concatenation, lacking the capability to adaptively filter information [22]. This indiscriminate fusion inevitably propagates background noise and environmental interference into the final feature representation, thereby suppressing the saliency of subtle thermal defects [23].

To address the aforementioned challenges, this paper proposes GHM-DEIM, an improved DEIM-based [24] framework. Firstly, to enhance feature extraction from low-contrast thermal images, we design a specialized architecture, termed Grouped Multi-scale Aggregation Attention Network (GMAANet). Unlike standard architectures, GMAANet incorporates an optimized channel distribution strategy with a heterogeneous split-transform-merge design. This feature extractor effectively expands the receptive field to capture global thermal gradients while suppressing local texture noise. Secondly, targeting the large scale variations of PV defects, we reconstruct the encoder by incorporating a Hypergraph-based Adaptive Correlation Enhancement (HyperACE) mechanism and a Full-Pipeline Aggregation-and-Distribution (FullPAD) paradigm [25]. These modules facilitate efficient information flow between shallow and deep layers, ensuring that both tiny hot spots and large-area failures are accurately represented. Finally, we replace the standard concatenation operations with a Modulation Fusion Module (MFM) [26]. Unlike simple linear superposition, MFM adaptively re-weights features from different channels, effectively filtering out environmental background interference and highlighting the saliency of thermal anomalies.

Our main contributions are as follows:

1. GHM-DEIM is proposed, an improved DEIM-based framework specifically designed for subtle and scale-variant thermal anomaly detection in photovoltaic UAV infrared imagery, providing an accurate and efficient solution for automated solar farm inspection.
2. GMAANet is developed as a specialized module that incorporates an optimized channel distribution strategy and a heterogeneous split-transform-merge design, overcoming the inherent limitations of standard CNNs in extracting discriminative features from low-contrast infrared thermal imagery.
3. An enhanced encoder is developed through the integration of the HyperACE and FullPAD paradigms. These components leverage high-order correlations to effectively address significant scale variations in PV defects.
4. The MFM is integrated in place of conventional concatenation, enabling adaptive feature re-weighting that suppresses environmental background interference and accentuates subtle thermal anomalies.

## 2. Related Work

### 2.1. CNN-Based PV Thermal Anomaly Detection

Deep learning-based approaches have gradually superseded traditional manual feature extraction, establishing CNN as the dominant paradigm for analyzing PV thermal imagery. In this domain, feature discriminability and localization precision are pivotal factors affecting detection performance. To this end, a large number of methods based on two-stage detectors have emerged to ensure high inspection reliability [27,28,29,30]. Particularly, Wang et al. [31] optimized the Region Proposal Network (RPN) within the Faster R-CNN framework to adapt to the variable aspect ratios of PV strings. Similarly, Li et al. [32] introduced a multi-scale feature pyramid network (FPN) to enhance the detection of small-sized thermal anomalies, effectively reducing the false negative rate in high-resolution infrared images.

However, these two-stage methods typically suffer from high computational latency, ignoring the real-time constraints required for UAV-based inspections. To solve this problem, one-stage detectors that unify bounding box regression and classification into a single network were proposed [33,34]. Methods such as SSD and the YOLO series significantly improved inference speed, making onboard deployment feasible. For instance, Karch et al. [35]. deployed a lightweight YOLOv8 model on edge devices, achieving a balance between speed and accuracy for large-scale solar farm monitoring.

Despite the improved efficiency brought by one-stage detectors, their performance in PV thermal inspection is still constrained by the inherent limitations of CNN backbones [36]. Due to the lack of color and rich textures in thermal images, defects often exhibit low contrast with the background, and although attention mechanisms such as Squeeze-and-Excitation (SE) [37] and Efficient Multi-Scale Attention (EMA) [38] have been introduced to enhance feature discriminability, these improvements remain limited by the local receptive fields of convolution. Moreover, the widespread use of NMS further causes missed detections in densely arranged PV arrays, thereby restricting the achievable accuracy in complex operation and maintenance scenarios.

### 2.2. Transformer-Based Object Detection

The introduction of Transformers has triggered a paradigm shift in object detection, moving away from dense anchor predictions toward end-to-end set prediction. Carion et al. [18] pioneered this direction with DETR, which utilized a global self-attention mechanism to model long-range dependencies, successfully eliminating the need for hand-crafted components like NMS. To address the slow convergence and high computational complexity of the original DETR, Zhu et al. [39] proposed Deformable DETR, which restricted attention to a small set of key sampling points around a reference, thereby enhancing multi-scale feature aggregation. Building on these foundations, Zhao et al. [19] developed RT-DETR, an efficient real-time architecture that employs a hybrid encoder to decouple intra-scale interaction and cross-scale fusion. Concurrently, D-FINE [40] further improved localization accuracy through refined boundary regression. Most recently, DEIM [24] has achieved leading performance by addressing the sparse supervision bottleneck inherent in the standard one-to-one (O2O) matching of DETR models. As an innovative training framework, DEIM introduces a Dense O2O matching strategy that significantly increases the number of positive samples through additional target incorporation. To mitigate the potential performance degradation from low-quality matches introduced by this dense strategy, it proposes the Matchability-Aware Loss (MAL), which optimizes matches across various quality levels to ensure stable and effective supervision. By synergizing these strategies, DEIM accelerates convergence and sets a new real-time detection benchmark by significantly boosting the performance of architectures like D-FINE.

Although DEIM achieves SoTA performance on general benchmarks, it was not engineered to exploit the unique spectral properties of thermal imagery, potentially limiting its adaptability to the intricate feature nuances and complex scene dynamics inherent to this domain. GHM-DEIM fills this gap by seamlessly integrating domain-specific mechanisms into the DEIM architecture, ensuring robust adaptation to the thermal domain.

## 3. Materials and Methods

### 3.1. Overall Framework

**Figure 1:**
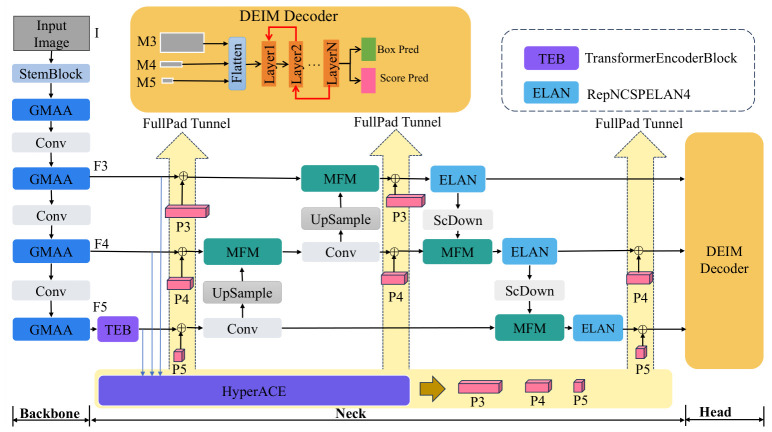
Overall architecture of the proposed PV thermal anomaly detection network.

As illustrated in Figure 1, GHM-DEIM is presented, a unified end-to-end framework based on an improved DEIM architecture to address the aforementioned challenges. Formally, given an input thermal image *I*, the processing pipeline begins with discriminative feature extraction. A specialized backbone is constructed by stacking GMAA. Through a heterogeneous split-transform-merge design with an optimized channel distribution strategy, this backbone effectively expands the receptive field to capture global thermal gradients, yielding multi-scale feature maps $F=\{F_{3},F_{4},F_{5}\}$. Subsequently, to handle large scale variations, these features are transformed by an encoder enhanced with HyperACE and FullPAD paradigm. This stage models complex non-local correlations to ensure accurate representation across all scales, resulting in context-aware features $P=\{P_{3},P_{4},P_{5}\}$.

Following the encoding phase, the MFM is introduced to replace standard concatenation operations. This module adaptively re-weights feature channels based on semantic importance to suppress environmental background interference, producing a refined feature set $M=\{M_{3},M_{4},M_{5}\}$. Finally, these modulated features are flattened and fed into a Transformer-based decoder, where a set of learnable object queries interacts with the image features to generate final predictions. The entire network is optimized using the advanced matching strategy and loss function derived from the DEIM paradigm, ensuring precise localization and rapid convergence for PV thermal anomaly detection.

### 3.2. GMAANet: Grouped Multi-Scale Aggregation Attention Network

The original DEIM-D-FINE framework utilizes High Performance GPU Network V2 (HGNetV2) as its backbone, which is constructed by stacking HGBlock. While HGBlock are highly effective in general visual tasks due to their dense connectivity and efficient gradient propagation, they exhibit limitations when applied to PV thermal anomaly detection. Specifically, the HGBlock relies predominantly on standard convolutional operations with fixed, local receptive fields [41]. In low-contrast thermal imagery, where defect signatures are often subtle and visually similar to environmental noise, this local processing paradigm fails to effectively model long-range dependencies. Consequently, HGBlock struggle to distinguish between localized hot spots and global thermal gradients, leading to potential false positives in complex backgrounds. To overcome these inherent structural drawbacks, we employ a series of GMAANet modules. Unlike the homogeneous processing in HGBlock, the GMAANet introduces a heterogeneous split-transform-merge architecture that explicitly synergizes local feature extraction with global context modeling, thereby enhancing feature discriminability without relying on the pure stacking of convolutions.

**Figure 2:**
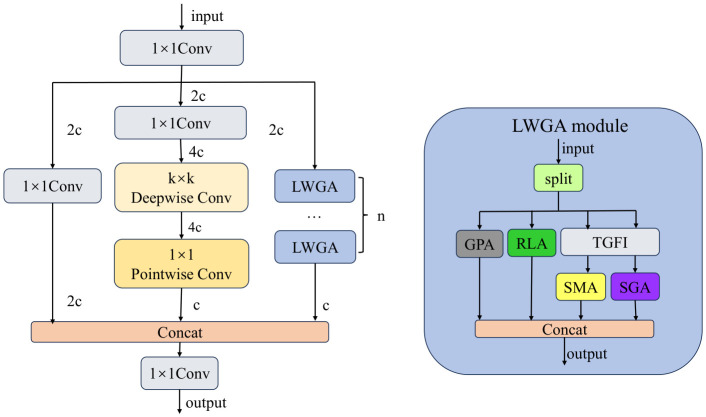
Structure of the GMAANet. It adopts a three-branch design to fuse identity, local, and global features.

The internal structure of the proposed GMAANet, as illustrated in Figure 2, is designed to process information at multiple granularities simultaneously. Given an input feature tensor $X\in R^{H\times W\times C}$, the block first expands the channel dimension to 2c via a 1×1 convolution to enrich the feature space. To address the redundancy often present in thermal data, this expanded feature set is then split along the channel dimension into three parallel branches: an identity branch, a local context branch, and a global context branch. The identity branch employs a simple 1×1 convolution to preserve primitive feature information and facilitate stable gradient flow, akin to a residual connection. The local context branch is specifically designed to capture high-frequency thermal details, such as the sharp edges of potential defects. It adopts an inverted residual bottleneck structure, where features are first expanded, processed by a k×k Depthwise Convolution to encode spatial details, and then projected back. This local transformation process can be mathematically formulated as:

(1) $$F_{\mathrm{local}}={\mathrm{PW}}_{\mathrm{proj}}\;\left(\sigma \;\left({\mathrm{DW}}_{k\times k}\;\left({\mathrm{PW}}_{\exp}\left(X_{\mathrm{mid}}\right)\right)\right)\right)$$

where PW denotes pointwise convolution, DW denotes depthwise convolution, and σ(·) represents the activation function.

To address the limitation of HGBlock in capturing global semantic information, The third branch incorporates a global context modeling module tasked with capturing long-range dependencies to differentiate true anomalies from background interference. The input features are further divided into four subgroups to perform specialized attention operations: Gate Point Attention (GPA), Regular Local Attention (RLA), Sparse Medium-range Attention (SMA), and Sparse Global Attention (SGA). This grouping strategy avoids the computational heaviness of full self-attention while ensuring diverse contextual modeling. Specifically, for the SMA and SGA groups, A Top-*k* Global Feature Interaction (TGFI) mechanism is adopted. Unlike standard global average pooling which might dilute small defect signals, TGFI selects only the most significant *k* semantic tokens to represent the global thermal distribution. The selection of these key semantic descriptors $K_{select}$ and values $V_{select}$ from the global feature space is defined as:

(2) $$K_{\mathrm{select}},V_{\mathrm{select}}={\mathrm{Top}}_{k}\left(K\right),{\mathrm{Top}}_{k}\left(V\right).$$

By interacting the local queries *Q* with these selected global descriptors, the network can focus on the most relevant thermal regions while suppressing irrelevant background noise. The sparse attention output for these groups is computed as:

(3) $$Y_{\mathrm{sparse}}=\mathrm{Softmax}\;\left(\frac{Q\cdot {\left(K_{\mathrm{select}}\right)}^{⊤}}{\sqrt{d_{k}}}\right)\cdot V_{\mathrm{select}}$$

where $d_{k}$ is the scaling factor. This mechanism ensures that the network possesses a global receptive field.

Finally, the outputs from the identity maintenance, local texture extraction, and global context modeling branches are aggregated to form a comprehensive feature representation. This multi-branch fusion allows the network to adaptively weigh the importance of local details versus global context depending on the input image characteristics. The features from all three branches are concatenated along the channel dimension and then fused via a final 1×1 convolution to mix the information and restore the channel dimensions. The overall output $Y_{out}$ of the GMAANet is expressed as:

(4) $$Y_{\mathrm{out}}=K_{1\times 1}\left(\mathrm{Concat}(F_{\mathrm{id}},F_{\mathrm{local}},F_{\mathrm{global}})\right).$$

By stacking these GMAANet hierarchically, the proposed hierarchical architecture generates a robust multi-scale feature pyramid $F=\{F_{3},F_{4},F_{5}\}$ that is inherently more discriminative for thermal anomaly detection than the standard representations produced by HGNetv2.

### 3.3. Hypergraph-Based Context Encoder

Although the GMAANet effectively extracts multi-level thermal features, the spatial distribution of PV defects presents a unique challenge: thermal anomalies often appear as disjoint clusters with significant scale variations. Standard CNN and FPN primarily model pairwise relationships between adjacent pixels, limiting their ability to capture high-order correlations among non-local defect patterns [42]. To address limitation, and inspired by hypergraph modeling and context-enhancement strategies, GHM-DEIM incorporates the HyperACE module and the FullPAD paradigm to construct an enhanced hypergraph-based context encoder. This encoder establishes non-local interactions in the thermal feature space to aggregate cross-channel and cross-location context, suppress redundancy, strengthen discriminative representations.

**Figure 3:**
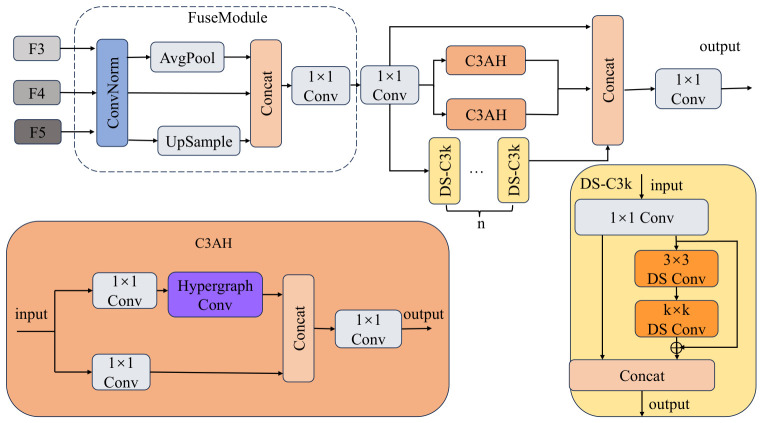
Schematic illustration of the Hypergraph-based Module. The module projects flattened feature maps into a latent space to construct a dynamic hypergraph.

As shown in Figure 3, The multi-scale feature maps $F=\{F_{3},F_{4},F_{5}\}$, obtained from the backbone, are first projected into a unified feature space. Let $X\in R^{N\times C}$ represent the flattened feature nodes. In contrast to static graphs, a dynamic construction of the hypergraph G=(V,E) is employed to capture multi-to-multi relationships. To avoid the computational complexity of all-pair comparisons, we introduce a set of learnable Hyperedge Prototypes $M\in R^{K\times C}$, which serve as cluster centers for semantic patterns (e.g., “high-temperature regions” or “shadow edges”).

The relationship between visual nodes and these prototypes is established via a subspace projection. Query embeddings *Q* and value embeddings *V* are generated as follows:

(5) $$Q=\phi_{q}\left(X\right),\;V=\phi_{v}\left(X\right)$$

where ϕ(·) denotes linear transformations. In contrast to a standard global attention map, the Node-to-Hyperedge Incidence Matrix $H\in R^{N\times K}$ is computed to represent the hypergraph structure. Specifically, the probability of the *i*-th pixel node belonging to the *j*-th hyperedge prototype is derived through a prototype-matching operation:

(6) $$H_{ij}=\frac{\exp(Q_{i}\cdot M_{j}^{T}/\sqrt{d})}{\sum_{k=1}^{K}\exp\left(Q_{i}\cdot M_{k}^{T}/\sqrt{d}\right)}.$$

This formulation effectively performs a soft clustering, assigning spatially disjoint but semantically similar defects to the same hyperedge.

Utilizing the incidence matrix *H*, feature propagation is performed based on spectral hypergraph theory. Node and hyperedge degree matrices are first defined to normalize the graph structure:

(7) $$D_{v}=\mathrm{diag}\left(\sum_{j=1}^{K}H_{ij}\right),\;D_{\varepsilon }=\mathrm{diag}\left(\sum_{i=1}^{N}H_{ij}\right).$$

The feature aggregation and distribution process is mathematically modeled as a two-stage message passing. First, node information is aggregated to hyperedges, and then the hyperedge context is broadcast back to nodes. This spectral convolution is formulated as:

(8) $$Z=D_{\nu }^{-1/2}HW_{\mathrm{agg}}D_{\varepsilon }^{-1/2}H^{⊤}D_{\nu }^{-1/2}V$$

where $W_{\mathrm{agg}}$ is the learnable aggregation weight. This operation allows a defect pixel to instantly access the context of all other pixels in the same hyperedge, regardless of distance. The encoded features are finalized via a residual update:

(9) $$X_{\mathrm{out}}=\mathrm{LayerNorm}\left(X+\mathrm{Dropout}\left(ZW_{\mathrm{out}}\right)\right).$$

Complementary to the encoder structure, we incorporate the FullPAD paradigm to address the scale variation of thermal defects. Unlike the sequential flow in FPN, FullPAD establishes three distinct distribution tunnels to inject the hypergraph-enhanced features $X_{\mathrm{out}}$ into different stages of the detection pipeline.

Let $T_{\mathrm{dist}}\left(\cdot \right)$ represent the distribution function that aligns the resolution of the global context $X_{\mathrm{out}}$ to a specific target scale *l*. The feature update rule for the *l*-th scale in the pipeline is defined as:

(10) $$F_{1}^{\mathrm{update}}=F_{1}+\lambda_{1}\cdot T_{\mathrm{dist}}\left(X_{\mathrm{out}},l\right)$$

where the alignment function $T_{\mathrm{dist}}$ typically involves adaptive upsampling or downsample:

(11) $$T_{\mathrm{dist}}\left(x,l\right)=\left\{\begin{array}{cc} \mathrm{Upsample}(x), & \mathrm{if}\;\mathrm{size}\left(x\right)<\mathrm{size}\left(F_{l}\right)\\ \mathrm{Downsample}(x), & \mathrm{if}\;\mathrm{size}\left(x\right)>\mathrm{size}\left(F_{l}\right) \end{array}\right.$$

By utilizing these tunnels, the network achieves a global-to-local feature propagation mechanism, ensuring that even the smallest defect proposal at the lowest pyramid level is informed by the global thermal topology captured by HyperACE. The final integrated feature set $F_{\mathrm{final}}$ serves as the robust input for the decoding head:

(12) $$F_{\mathrm{final}}=\left\{{\mathrm{Conv}}_{1\times 1}\left(\left[F_{l}^{\mathrm{update}},P_{l}\right]\right)|l\in \left\{3,4,5\right\}\right\}.$$

### 3.4. Adaptive Feature Integration via MFM

**Figure 4:**
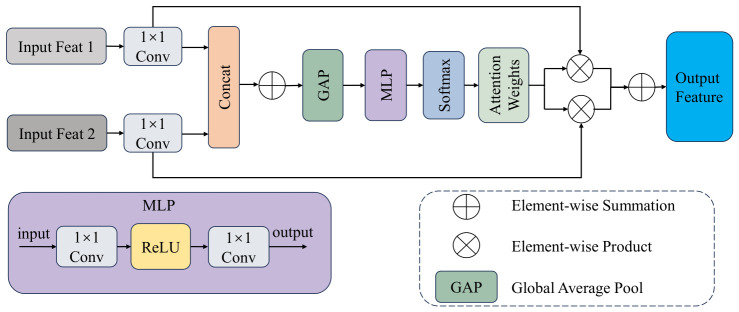
Structure of the MFM. It adaptively integrates multi-scale features by learning branch-specific attention weights through global spatial statistics, effectively enhancing discriminative thermal anomaly patterns while suppressing background noise.

Following the global topology modeling by the Hypergraph-based Context Encoder, it is crucial to effectively fuse these globally enhanced features with the local hierarchical representations from the backbone. Standard feature integration typically relies on simple concatenation or element-wise addition. However, these operations treat all feature channels equally, which may dilute the highly discriminative thermal anomaly patterns with background noise or redundant textures. To address this, we introduce the MFM to replace conventional concatenation, acting as an adaptive gate for feature integration. The detailed architecture of the proposed MFM is illustrated in Figure 4.

Given *N* input feature streams denoted as $\left\{F_{1},F_{2},\ldots ,F_{N}\right\}$, the MFM module first employs 1×1 convolutions to project them into a unified channel dimension *C*, obtaining the aligned features ${\hat{F}}_{i}$:

(13) $${\hat{F}}_{i}={\mathrm{Conv}}_{1\times 1}\left(F_{i}\right),\;i\in \left\{1,2,\ldots ,N\right\}.$$

To capture the collaborative global context across all feature streams, these aligned features are element-wise summed. The aggregated feature map is then squeezed through a Global Average Pooling ($H_{\mathrm{GAP}}$) operation to generate a channel-wise global descriptor $z\in R^{C\times 1\times 1}$:

(14) $$z=H_{\mathrm{GAP}}\left(\sum_{i=1}^{N}{\hat{F}}_{i}\right).$$

This descriptor encapsulates the holistic statistics of the fused representations. Subsequently, a Multi-Layer Perceptron (MLP) block—consisting of two 1×1 convolutions separated by a ReLU activation—is utilized to learn the non-linear cross-channel interactions. The output is reshaped and passed through a Softmax function along the stream dimension to generate the adaptive attention weights A:

(15) A=Softmax(MLP(z))

where $A\in R^{N\times C\times 1\times 1}$ can be decomposed into a set of weight vectors $\left\{A_{1},A_{2},\ldots ,A_{N}\right\}$. The Softmax operation introduces a competitive mechanism among the different streams, forcing the network to select the most discriminative channels.

Finally, the attention weights are broadcasted and element-wise multiplied with their respective aligned features, followed by a summation across all streams to yield the integrated output $F_{\mathrm{out}}$:

(16) $$F_{\mathrm{out}}=\sum_{i=1}^{N}A_{i}⊗{\hat{F}}_{i}$$

where ⊗ denotes element-wise multiplication along the channel dimension. By explicitly modulating the features, MFM acts as an information filter, enhancing salient thermal defect regions while suppressing irrelevant background interference, thus providing highly refined integrated features for the final detection head.

## 4. Experiments and Results

### 4.1. Dataset

To evaluate the performance of the GHM-DEIM in PV thermal anomaly detection, we conduct extensive experiments on two datasets: the ThermoSolar-PV dataset [43] and the PV-HSD-2025 dataset [44].

The ThermoSolar-PV dataset is curated for anomaly detection in PV modules using thermal imagery, providing a realistic benchmark for defect recognition. It consists of a diverse set of infrared thermal images collected under various real-world operating conditions. The dataset is categorized into eight distinct classes: Single Hotspot, Multi Hotspots, Single Diode, Multi Diode, Single Bypassed Substring, Multi Bypassed Substring, String (Open Circuit), and String (Reversed Polarity).

The PV-HSD-2025 dataset is specifically designed for the hotspots. It provides a diverse collection of thermal imagery where the spatial characteristics and scales of the annotated instances align with real-world industrial inspection protocols.

In our experiments, both datasets are randomly partitioned into training, validation, and testing sets with a ratio of 8:1:1. The ThermoSolar-PV dataset is divided into 5871 images for training, 734 for validation, and 734 for testing. Similarly, the PV-HSD-2025 dataset is divided into 3220 training, 402 validation, and 403 testing images. All experiments are conducted on these fixed data splits to ensure the reproducibility and fairness of the results.

The pixel intensities of the infrared thermal images in both datasets are proportional to surface temperature. As absolute temperature calibration data are not provided by the original dataset sources, all thermal images in this study are used solely for anomaly localization and defect pattern recognition, rather than for quantitative temperature analysis.

### 4.2. Evaluation Metrics

To quantitatively and comprehensively evaluate the performance of GHM-DEIM in detecting thermal anomalies, we employ several standard object detection metrics, including Precision (*P*), Recall (*R*), and mean Average Precision (mAP). Precision measures the ratio of true positive detections to all positive predictions, reflecting the model’s ability to suppress false alarms triggered by environmental background interference. Recall indicates the proportion of correctly identified anomalies out of all ground-truth samples, which is crucial for ensuring that critical PV module failures are not overlooked. These metrics are defined as follows:

(17) $$P=\frac{\mathrm{TP}}{\mathrm{TP}+\mathrm{FP}},\;R=\frac{\mathrm{TP}}{\mathrm{TP}+\mathrm{FN}}$$

where TP, FP, and FN represent true positives, false positives, and false negatives, respectively.

Furthermore, mAP is utilized as the primary indicator for evaluating the overall detection accuracy across all categories. It is calculated by averaging the Area Under the Precision-Recall Curve (AP) for each anomaly class:

(18) $$\mathrm{mAP}=\frac{1}{N}\sum_{i=1}^{N}\int_{0}^{1}P_{i}\left(R_{i}\right)dR_{i}$$

where *N* denotes the total number of anomaly categories. Specifically, we report mAP@50% to assess general detection effectiveness, and mAP@50–95% to evaluate the precision of the predicted bounding box localization. Together, these metrics offer a robust framework for assessing the model’s efficacy in complex solar farm environments.

### 4.3. Implementation Details

GHM-DEIM is implemented using the PyTorch 2.8.0 framework and trained on a workstation equipped with an Intel Core i7-14700KF CPU and an NVIDIA GeForce RTX 5060 Ti GPU. The training process is accelerated using CUDA 12.9 to fully utilize the GPU computing capability. To ensure optimal convergence and robustness, we adopt a DEIM-inspired training schedule spanning 200 epochs. This strategy is characterized by a three-stage augmentation pipeline designed to align the model with the complex distribution of PV thermal anomalies. Specifically, during the initial 20 epochs, data augmentation is disabled to allow the network to establish stable early-stage representations from the raw thermal signatures. From epoch 20 to 180, high-intensity augmentations—including Mosaic, Mixup, and random perspective transformations—are fully activated to enhance the model’s ability to handle scale variations and disjoint defect clusters. In the final 20 epochs (180–200), these augmentations are closed to facilitate fine-tuning on the real-world image distribution, ensuring the high-precision localization of thermal bounding boxes. The detailed hyperparameter configurations are summarized in Table 1.

### 4.4. Comparative Experiment

To evaluate GHM-DEIM in thermal anomaly detection, we conduct comparative experiments against baseline object detectors on the ThermoSolar-PV and PV-HSD-2025 datasets. The selected baselines encompass CNN-based architectures namely YOLOv5 [45], YOLOv8 [46], YOLOv12 [47], and YOLOv13 [25]; transformer-based detectors including RT-DETR [19], D-FINE [40], and DEIM-D-FINE [24]; and thermal-specific models namely MS-YOLO [48] and GM-DETR [49]. All models are trained from scratch using identical dataset partitions and configurations to ensure fair comparison.

The quantitative results summarized in Table 2 for the ThermoSolar-PV dataset demonstrate that GHM-DEIM achieves the highest performance across all evaluation metrics, reaching 88.6% mAP@50% and 72.5% mAP@50–95%. This performance represents an absolute improvement of 4.7% over the DEIM-D-FINE-S baseline. Other evaluated detectors, including the CNN-based YOLO series, transformer-based models and thermal-specific models namely MS-YOLO and GM-DETR, yield lower accuracy with mAP@50% values ranging from 61.6% to 80.8%. Furthermore, our approach maintains a balance between Precision and Recall at 87.2% and 84.7% respectively. Regarding efficiency, the model operates at 116.1 FPS using 13.5 million parameters and 28.8 GFLOPs, exceeding the inference speeds of DEIM-D-FINE-S and RT-DETR-R18. Although MS-YOLO and YOLOv8s exhibit higher frame rates at 156.1 FPS and 131.5 FPS, their detection accuracy remains significantly lower than our method. These data confirm that GHM-DEIM secures a superior balance between detection capability and computational speed.

Table 3 presents the experimental results on the PV-HSD-2025 dataset. GHM-DEIM reaches 74.2% mAP@50% and 31.8% mAP@50–95%, which constitutes a 1.8% and 0.7% increase respectively compared to the DEIM-D-FINE-S baseline. In contrast, the mAP@50% values for CNN-based and transformer-based detectors remain below 68.7%, with YOLO series models ranging from 66.0% to 68.2%. The thermal-specific models MS-YOLO and GM-DETR yield mAP@50% scores that are 32.7% and 9.9% lower than GHM-DEIM. Regarding inference efficiency, the network operates at 153.4 FPS, which is 47.7 FPS faster than DEIM-D-FINE-S and 17.3 FPS faster than RT-DETR-R18. While YOLOv13s achieves a higher frame rate of 173.9 FPS, its mAP@50% is 7.5% lower than our results.

**Figure 5:**
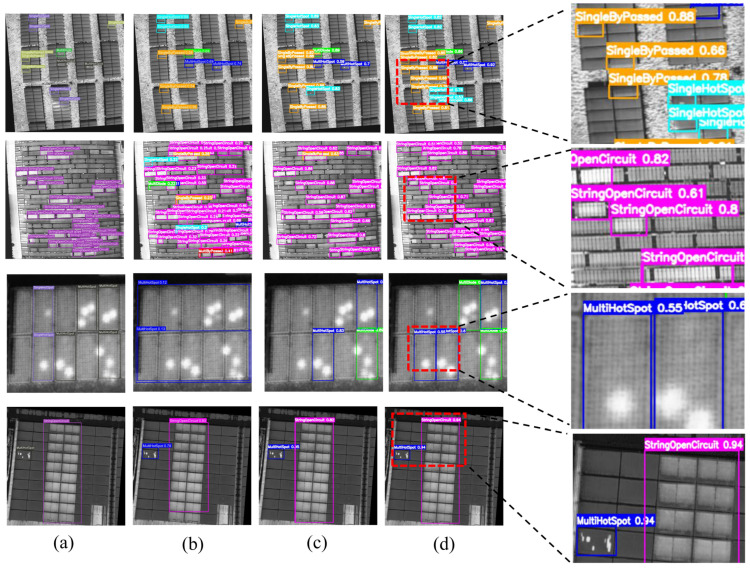
Qualitative comparison of detection results under various challenging thermal PV scenarios. (**a**) Ground Truth annotations. (**b**) Detection results of YOLOv13s. (**c**) Detection results of the baseline DEIM-D-FINE-S. (**d**) Detection results of GHM-DEIM. From top to bottom, the rows represent: multi-class anomaly co-occurrence, dense anomaly clusters, medium-to-large irregular defects, and cross-scale target detection. Detection boxes are color-coded by defect category: pink = String (Open Circuit), orange = Single Bypassed Substring, blue = Multi Hotspot, green = Multi Diode. Red dashed boxes indicate the enlarged regions shown on the right for detailed inspection of detection labels and confidence scores.

Figure 5 shows the qualitative comparison of detection results under four representative scenarios: multi-class anomalies, dense targets, medium-to-large defects, and cross-scale detection. The detection boxes are color-coded by defect category: pink for String (Open Circuit), orange for Single Bypassed Substring, blue for Multi Hotspot, and green for Multi Diode. The red dashed boxes in column (d) indicate representative regions, which are enlarged on the right side of the figure for detailed inspection.

In Row 1 (multi-class anomaly co-occurrence), the scene contains four defect categories simultaneously: Single Hotspot, Multi Hotspots, Single Bypassed Substring, and Multi Diode. As shown in column (b), YOLOv13s produces several false positives and fails to localize the Single Bypassed instances in the upper region. Column (c) shows that DEIM-D-FINE-S completely misses the Multi Diode anomalies. In contrast, GHM-DEIM in column (d) successfully detects all four defect categories, with the confidence score for the Single Hotspot instances reaching 0.88.

In Row 2 (dense anomaly clusters), the scene is dominated by densely arranged String defects distributed across the entire PV array. As shown in columns (b) and (c), both YOLOv13s and DEIM-D-FINE-S exhibit missed detections and false positives throughout the array. GHM-DEIM in column (d) produces more complete detections, with confidence scores of around 0.82.

In Row 3 (medium-to-large irregular defects), the scene contains Multi Hotspot anomalies, which appear as distinct bright high-temperature regions. As shown in column (b), YOLOv13s detects these anomalies with low confidence scores of 0.12 and 0.13. Column (c) shows that DEIM-D-FINE-S detects only one region with a confidence score of 0.83, missing the smaller hotspot instances on the right side. GHM-DEIM in column (d) correctly localizes all hotspot instances, including the Multi Diode anomaly in the upper-right corner.

In Row 4 (cross-scale detection), the scene includes both large-scale String (Open Circuit) defects covering entire panel strings and small-scale Multi Hotspot anomalies in the lower-left corner. As shown in column (b), YOLOv13s detects the String (Open Circuit) with a confidence score of 0.83 but fails to detect the small-scale Multi Hotspot. Column (c) shows that DEIM-D-FINE-S detects the String (Open Circuit) with a confidence score of 0.82, but similarly misses the small-scale target. GHM-DEIM in column (d) achieves the highest confidence score of 0.94 for the String (Open Circuit) and simultaneously detects the small-scale Multi Hotspot with a confidence score of 0.94, which verifies that GHM-DEIM bridges the gap between large-scale structural anomaly recognition and small-scale thermal defect localization.

### 4.5. Ablation Experiment

To evaluate the specific contributions of the GMAANet, the Hypergraph-based Context Encoder, and the Modulation Fusion Module, we conduct ablation experiments on the ThermoSolar-PV and PV-HSD-2025 datasets. The DEIM-D-FINE model is established as the baseline architecture. Each proposed component is integrated into this baseline incrementally to verify the resulting performance gains. To ensure experimental consistency and eliminate training bias, all architectural variants follow the identical DEIM-based schedule and hyperparameter configurations. The evaluation records variations in detection accuracy and physical complexity across both datasets to quantify the efficacy of the individual architectural modules.

Table 4 presents the ablation results on the ThermoSolar-PV dataset, and Table 5 summarizes the corresponding performance on the PV-HSD-2025 dataset. These evaluations detail the influence of the GMAANet, the Hypergraph-based Encoder, and the Modulation Fusion Module on the detection metrics across both environments.

#### 4.5.1. GMAANet

The integration of the Grouped Multi-scale Aggregation Attention Network, denoted as GMAA, into the baseline architecture improves detection accuracy across both datasets. On the ThermoSolar-PV dataset, substituting the original backbone with the GMAANet increases the mAP@50% from 83.9% to 86.1% and the mAP@50–95% from 67.8% to 69.4%, yielding absolute gains of 2.2% and 1.6% respectively. Parallel evaluations on the PV-HSD-2025 dataset show an mAP@50% increase of 1.1% and an mAP@50–95% increase of 0.8% compared to the baseline. These performance enhancements correspond to an increase in physical complexity, adding 2.5 million parameters and 3.7 GFLOPs to the original network.

**Figure 6:**
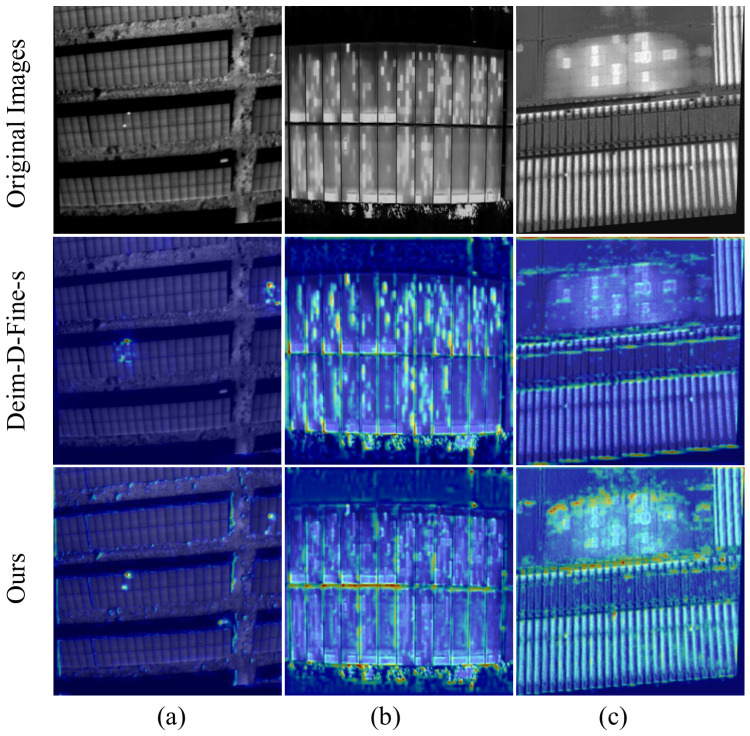
Visualization of feature activation heatmaps. (**a**) Small-scale thermal anomalies; (**b**) Dense anomaly clusters; (**c**) Irregular clustered anomalies. The top, middle, and bottom rows correspond to the original images, HG_Stage heatmaps, and GMAANet heatmaps, respectively.

To further investigate the internal interpretability of our architectural refinements, we utilize Gradient-weighted Class Activation Mapping++ (Grad-CAM++) [50] to visualize the feature response patterns. Figure 6 illustrates a 3 × 3 comparative grid across three representative scenarios: small-scale anomalies (Column a), dense thermal defects (Column b), and irregular clustered anomalies (Column c). Compared to the HGNetV2, which often exhibits diffuse activation and susceptibility to background noise, replacing it with the GMAANet module yields significantly more concentrated and structurally coherent heatmaps. In small-scale scenarios, GMAANet precisely isolates subtle thermal signatures by suppressing irrelevant peripheral artifacts. For dense and clustered defects, it generates intense, geometrically aligned activations that accurately map the actual distribution of the anomalies rather than producing smeared or fragmented responses. These visualizations compellingly demonstrate that the grouped multi-scale aggregation mechanism effectively balances fine-grained detail extraction with global contextual awareness, ensuring robust defect perception against complex industrial backgrounds.

#### 4.5.2. Hypergraph-Based Encoder

The ablation results on the ThermoSolar-PV and PV-HSD-2025 datasets demonstrate the contribution of HyperACE to the encoder. When HyperACE is incorporated into the baseline, mAP@50% reaches 86.7% and mAP@50–95% reaches 69.3% on the ThermoSolar-PV dataset, while mAP@50% reaches 72.7% and mAP@50–95% reaches 31.3% on the PV-HSD-2025 dataset, with 11.4 M parameters and 27.1 GFLOPs. The Hypergraph-based encoder delivers absolute improvements of 2.8% mAP@50% and 1.5% mAP@50–95% on the ThermoSolar-PV dataset and 1.4% mAP@50% and 1.0% mAP@50–95% on the PV-HSD-2025 dataset over the baseline for a net increase of 1.2 M parameters.

**Figure 7:**
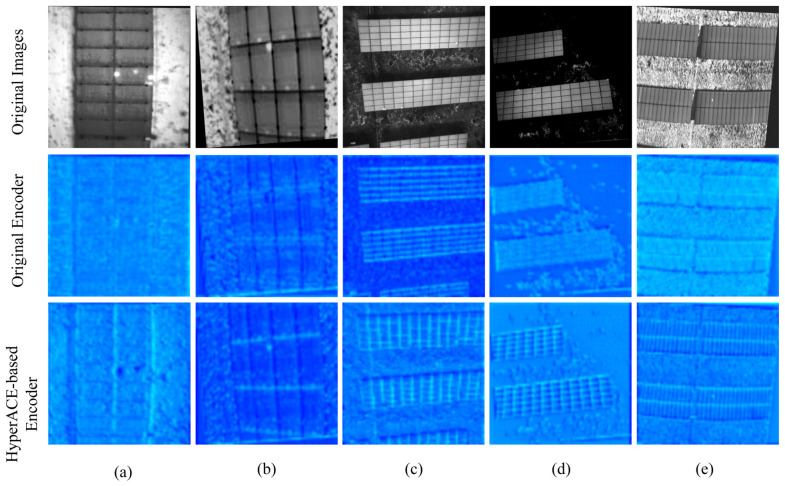
Feature map visualization for the original and Hypergraph-based encoders under five typical scenarios (**a**–**e**). The Hypergraph-based encoder exhibits higher structural coherence and clearer semantic boundaries in identifying PV module arrangements.

As shown in the Figure 7, the feature maps produced by the Hypergraph-based encoder differ from those of the original encoder across the five samples (a)–(e). In samples (a) and (b), the Hypergraph-based encoder retains the vertical grid structures of the solar panels, whereas the original encoder produces dispersed activation patterns. In samples (c), (d), and (e), the Hypergraph-based encoder captures the horizontal boundaries and overall panel arrangements, while the original encoder does not preserve these structural details. The visualization indicates that HyperACE enables the encoder to extract key semantic features from the input images more effectively, which is consistent with the detection performance gains reported above.

#### 4.5.3. MFM

The last part of the ablation study examines the effect of MFM. When MFM is added to the baseline alone, the mAP@50% reaches 86.9% on ThermoSolar-PV and 72.5% on PV-HSD-2025, corresponding to gains of 3.0% and 1.2%, respectively. At the same time, the number of parameters decreases from 10.2M to 9.75M, and the computational cost is reduced from 25.0 to 22.2 GFLOPs. These results show that replacing the original fusion operation with MFM brings a clear efficiency benefit while maintaining stronger feature representation. The fact that MFM alone slightly surpasses HyperACE alone on ThermoSolar-PV is also understandable from the structure of the original HGNetV2-based baseline. HGNetV2 mainly depends on convolutional feature extraction and concatenation-style feature aggregation. In this case, redundant responses and background-sensitive information may still be passed to the neck. MFM acts exactly on this fusion stage, so it can directly re-weight different feature streams and improve the quality of the fused representation. HyperACE, by comparison, is inserted into the encoder and aims to model high-order non-local relationships. Its effect is more dependent on whether the input features are already sufficiently discriminative. Therefore, the better standalone result of HGNetV2+MFM does not mean that MFM has the same function as HyperACE; rather, it shows that MFM more directly addresses the fusion weakness of the original baseline.

The result of GMAANet+MFM further shows that the interaction between modules is not simply additive. GMAANet has already introduced attention-based feature selection in the backbone, while MFM performs another round of adaptive re-weighting during feature fusion. When these two modules are connected directly, without the contextual modeling provided by HyperACE, the re-weighting process may become too selective. Some strong thermal responses can be emphasized, but weak anomaly cues, which are important for subtle defect detection, may be suppressed at the same time. This provides a possible explanation for the “1 + 1 < 2” behavior of the GMAANet+MFM setting. After HyperACE is introduced between them, the feature streams are first reorganized and enhanced through hypergraph-based non-local context modeling. MFM then receives features with more consistent semantic context, which makes its fusion process more reliable. This also explains why GMAANet+HyperACE already obtains 88.1% mAP@50%, and the full model further improves the result to 88.6%. In this setting, MFM should be viewed as a complementary fusion and efficiency-refinement module, while the main representation improvement comes from the coupled GMAANet and HyperACE pipeline.

## 5. Conclusions

This paper presents GHM-DEIM, a thermal anomaly detection framework built upon the DEIM architecture for PV module infrared inspection. To address the challenges posed by low target contrast, large scale variations, and subtle thermal defects in infrared imagery, several dedicated modules were incorporated into the network. Experimental results demonstrate that GMAANet preserves more informative features under low-contrast conditions, thereby enhancing feature representation capability. By introducing the HyperACE encoder, the model is able to capture long-range topological dependencies more effectively, leading to improved detection performance for anomalies of varying scales. In addition, the MFM module strengthens responses to weak anomalies while maintaining relatively low computational overhead.

Experiments conducted on the ThermoSolar-PV and PV-HSD-2025 datasets further verify the effectiveness of the proposed method. Compared with representative detectors such as YOLOv13 and DEIM-D-FINE-S, GHM-DEIM achieves improvements of 4.7% and 2.9% in mAP@50%, respectively. Visual analysis of the detection results also shows that the proposed model can localize thermal anomaly regions more accurately and generate clearer feature responses.

Although promising results have been achieved, there is still room for further improvement. Future work will focus on network lightweighting and model compression techniques to support real-time deployment on resource-constrained edge devices. In addition, more diverse thermal infrared datasets from different environments and application scenarios will be incorporated to further improve the model’s generalization ability and robustness under varying climatic conditions and PV installation settings. 

## Figures and Tables

**Table 1:** Summary of experimental settings and hyperparameters.

Hyperparameter	Setting
Epoch	200
Image Size	640 × 640
Batch Size	4
Initial Learning Rate	0.0002
Weight Decay	0.0001
Optimizer	AdamW
Augmentation Schedule	20/180/20

**Table 2:** Quantitative comparison with representative object detection models on the ThermoSolar-PV dataset. Bold values indicate the best performance in each column.

Method	Precision (%)	Recall (%)	mAP@50 (%)	mAP@50–95 (%)	Params (M)	GFLOPs	FPS
YOLOv5s	65.5	70.7	71.0	48.5	9.1	24.1	87.0
YOLOv8s	76.5	76.9	80.8	56.1	11.1	28.7	131.5
YOLOv12s	77.7	73.4	79.8	57.6	9.1	19.6	88.5
YOLOv13s	74.9	73.9	77.6	54.2	9.0	21.0	73.2
MS-YOLO	61.9	63.6	61.6	40.4	**3.9**	**6.8**	**156.1**
RT-DETR-R18	82.5	75.5	77.7	58.0	20.0	60.0	92.6
GM-DETR	75.8	72.1	68.8	54.4	38.3	65.4	49.8
D-FINE-S	72.7	78.7	78.8	63.8	10.2	25.0	114.8
DEIM-D-FINE-S	78.9	78.8	83.9	67.8	10.2	25.0	106.2
**GHM-DEIM**	**87.2**	**84.7**	**88.6**	**72.5**	13.5	28.8	116.1

**Table 3:** Quantitative comparison with representative object detection models on the PV-HSD-2025 dataset. Bold values indicate the best performance in each column.

Method	Precision (%)	Recall (%)	mAP@50 (%)	mAP@50–95 (%)	Params (M)	GFLOPs	FPS
YOLOv5s	69.4	65.4	68.1	29.7	9.1	24.1	86.3
YOLOv8s	67.1	66.8	68.2	29.5	11.1	28.7	112.4
YOLOv12s	67.4	63.0	66.0	28.5	9.1	19.6	134.4
YOLOv13s	68.0	63.1	66.7	29.2	9.0	21.0	**173.9**
MS-YOLO	49.2	43.3	41.5	14.5	**3.9**	**6.8**	141.2
RT-DETR-R18	61.6	55.8	54.6	21.4	20.0	60.0	136.1
GM-DETR	66.7	59.1	64.3	24.6	38.3	65.4	50.7
D-FINE-S	68.6	69.7	68.7	28.8	10.2	25.0	136.9
DEIM-D-FINE-S	69.3	72.2	71.3	31.1	10.2	25.0	105.7
**GHM-DEIM**	**71.2**	**73.7**	**74.2**	**31.8**	13.5	28.8	153.4

**Table 4:** Progressive ablation study based on the DEIM-D-Fine-S framework on the ThermoSolar-PV dataset. Bold values indicate the results of the complete GHM-DEIM model.

DEIM-D-Fine-S	GMAANet	HyperACE	MFM	mAP@50 (%)	mAP@50–95 (%)	Params (M)	GFLOPs
√				83.9	67.8	10.2	25.0
√	√			86.1	69.4	12.7	28.7
√		√		86.7	69.3	11.4	27.1
√			√	86.9	70.6	9.75	22.2
√	√	√		88.1	72.0	13.9	31.4
√		√	√	87.0	70.0	12.4	27.4
√	√		√	85.9	68.7	12.2	26.2
√	√	√	√	**88.6**	**72.5**	**13.5**	**28.8**

**Table 5:** Progressive ablation study based on the DEIM-D-Fine-S framework on the PV-HSD-2025 dataset. Bold values indicate the results of the complete GHM-DEIM model.

DEIM-D-Fine-S	GMAANet	HyperACE	MFM	mAP@50 (%)	mAP@50–95 (%)	Params (M)	GFLOPs
√				71.3	30.3	10.2	25.0
√	√			72.4	31.1	12.7	28.7
√		√		72.7	31.3	11.4	27.1
√			√	72.5	31.6	9.75	22.2
√	√	√		72.0	31.6	13.9	31.4
√		√	√	72.1	31.0	12.4	27.4
√	√		√	72.2	31.7	12.2	26.2
√	√	√	√	**74.2**	**31.8**	**13.5**	**28.8**

## Data Availability

We clarify that our research findings are based on the analysis of publicly available datasets: ThermoSolar-PV: https://github.com/P-Darabi/SolarPanelsDefectDetector (accessed on 5 February 2026). PV-HSD-2025: https://universe.roboflow.com/misaka-cpn7n/pv-hsd-2025-orgxy (accessed on 6 February 2026).
